# Angiotensin II type 1 and type 2 receptors modulate TWIK1 channel expression and pain sensitivity in a rat model of neuropathic pain

**DOI:** 10.3389/fphar.2026.1833813

**Published:** 2026-05-21

**Authors:** Emanuel D. Peralta, Yanaysis Stable García, Santiago V. Soria, Sergio G. Benitez, Cristian G. Acosta

**Affiliations:** Laboratory of Neurobiological Studies, Instituto de Histología Y Embriología de Mendoza (IHEM), National Research Council of Argentina (CONICET) and Cuyo National University (UNCuyo), Mendoza, Argentina

**Keywords:** angiotensin-II, dorsal root ganglia, inflammatory cytokines, leak potassium channel, neuropathic pain, Twik1

## Abstract

**Background:**

The renin–angiotensin system (RAS) contributes to inflammatory and neuropathic pain, but whether its primary receptors, AT1R and AT2R, regulate the K2P channel TWIK1 in sensory neurons remains unknown. We investigated whether Ang-II receptors modulate TWIK1 expression and whether this pathway influences neuropathic pain.

**Methods:**

Adult female rats underwent chronic constriction injury (CCI) of the sciatic nerve and received continuous subcutaneous administration of vehicle, telmisartan (AT1R antagonist), PD123319 (AT2R antagonist), or their combination for 14 days. TWIK1, AT1R, and AT2R expression were analyzed in dorsal root ganglia (DRG) by immunohistochemistry and RT-qPCR. Behavioral outcomes included spontaneous foot lifting, cold allodynia, and mechanical hypersensitivity. Plasma cytokines were quantified by ELISA. Satellite glial cell (SGC) activation was assessed via GFAP immunoreactivity. *In vitro* DRG cultures were exposed to inflammatory stimuli to evaluate TWIK1 transcriptional regulation.

**Results:**

CCI reduced TWIK1 expression in DRG neurons, accompanied by dynamic regulation of AT1R and AT2R. Pharmacological blockade, particularly combined receptor inhibition, restored or increased TWIK1 expression, attenuated mechanical and cold hypersensitivity, reduced circulating pro-inflammatory cytokines (TNF-α, IL-1β, IL-6), and decreased SGC activation. Inflammatory stimulation *in vitro* reduced TWIK1 mRNA, supporting cytokine-mediated regulation.

**Conclusion:**

AT1R and AT2R regulate TWIK1 expression in sensory neurons through an integrated mechanism involving inflammatory signaling and glial activation. Dual receptor blockade produces the most consistent molecular and behavioral effects, supporting RAS modulation as a potential strategy for neuropathic pain management.

## 1 Introduction

Chronic pain is a prevalent and disabling condition resulting from sustained injury or inflammation of peripheral nerves and their target tissues (Abd-Elsayed et al., 2024; Cohen and Mao, 2014). Current treatments are insufficient: only about half of patients respond adequately, and nearly 80% report dissatisfaction (Geurts et al., 2017; Giovannini et al., 2021). This therapeutic gap largely reflects an incomplete understanding of the molecular and cellular mechanisms underlying persistent pain (Balzani et al., 2021; Yekkirala et al., 2017). Chronic pain is associated with altered excitability of primary sensory neurons, characterized by changes in resting membrane potential (Em), firing threshold, and action potential dynamics (Djouhri et al., 2012; Du et al., 2014; Lawson et al., 2019).

Components of the renin–angiotensin system (RAS) have emerged as modulators of nociceptor function. Inhibition of renin, angiotensin-converting enzyme (ACE), or Angiotensin-II type 1 and type 2 receptors (AT1R and AT2R) attenuates several pain conditions, including migraine, neuropathic, and acute inflammatory pain (Bali et al., 2014; Balogh et al., 2021; Costa et al., 2014). AT1R and AT2R are GPCRs widely expressed in neural and non-neural tissues (de Gasparo et al., 2000; Ziaja et al., 2021) and are present in DRG neurons (Benitez et al., 2017; Lenkei et al., 1997; Pavel et al., 2008). We have shown that they co-localize in nociceptors projecting to laminae I–II of the spinal cord and jointly regulate inflammation-induced cutaneous hyperinnervation (Benitez et al., 2020). AT1R blockade has anti-inflammatory and analgesic effects in rheumatoid arthritis and possibly in other pain states (Bayar et al., 2015; Chang and Wei, 2015; Dagenais and Jamali, 2005). Peripheral AT2R antagonism alleviates post-herpetic neuralgia (Rice et al., 2014), inflammatory and neuropathic pain (Anand et al., 2015; Chakrabarty et al., 2018; Chakrabarty et al., 2013; Shepherd and Mohapatra, 2018; Smith et al., 2013). Conversely, AT2R activation by mycolactone produces profound analgesia in Buruli ulcer, possibly via recruitment of two-pore domain (K2P) channels (Marion et al., 2014; Smith and Muralidharan, 2015).

K2P channels generate background K^+^ leak currents that stabilize the resting membrane potential and regulate neuronal excitability. These constitutively active, voltage-independent channels respond to diverse physical and chemical cues (Enyedi and Czirjak, 2010; Plant, 2012). Their inhibition depolarizes sensory neurons and amplifies pain, whereas their activation hyperpolarizes neurons and suppresses excitability (Acosta et al., 2014; Busserolles et al., 2016; Gada and Plant, 2019). Several K2P members have been implicated in nociception (Luo et al., 2021; Mathie et al., 2021).

TWIK1 (tandem of pore domains in a weak inward rectifying K^+^ channel, also known as K2P1/KCNK1) was the first cloned K2P channel (Lesage et al., 1996) and is widely expressed in multiple tissues, including the nervous system (Lesage et al., 1997; Luo et al., 2021; Talley et al., 2001). Experimental evidence indicates that TWIK1 contributes to neuronal excitability, astrocytic passive conductance, and glutamate release (Cho et al., 2018). Its activation by serotonin hyperpolarizes neurons and reduces excitability (Deng et al., 2007), whereas downregulation increases firing (Yarishkin et al., 2014). TWIK1 also forms heteromers with other K2P isotypes including TREK1, TASK1, and TASK3 (Khoubza et al., 2021). Importantly, TWIK1 is present in DRG neurons and has been implicated in neuropathic (Mao et al., 2017; Pollema-Mays et al., 2013) and chemotherapy-induced cancer pain (Jia et al., 2022; Mao et al., 2019).

Until recently, whether TWIK1 is regulated by renin–angiotensin signaling in sensory neurons remained unknown. However, we found that acute *in vitro* administration of Ang-II transiently upregulated TWIK1 in cultured rat DRG neurons, whereas *in vivo*, intradermal injection of the peptide resulted in a significant but moderate mechanical hypersensitivity. This event coincided with a lower expression of the channel in A-fibre like DRG neurons (Peralta et al., 2026). Still, a potential link between AT1R or AT2R activity and TWIK1 expression or function in pathological pain has not been investigated.

Here, we tested the hypothesis that AT1R and AT2R signaling modulates nociceptor excitability by regulating TWIK1 expression in DRG neurons. Using *in vivo* pharmacological manipulation, behavioral testing, RT-qPCR, ELISA, and quantitative immunohistochemistry, we examined whether selective modulation of AT1R or AT2R alters TWIK1 expression and alleviates neuropathic pain after unilateral chronic constriction injury of the sciatic nerve (CCI). We also assessed whether prolonged receptor antagonism affects AT1R or AT2R expression, a previously unexplored component of RAS-mediated pain modulation. By identifying TWIK1 as a potential downstream effector of AT1R and AT2R signaling in sensory neurons, this study provides new insight into the mechanisms linking RAS activity to nociceptor excitability and explores these receptors as potential therapeutic targets for refractory pathological pain.

## 2 Materials and methods

### 2.1 Experimental animals

For experiments, 3 to 6-month-old female Wistar rats (140–160 g; RRID:RGD_737929) were used throughout. Rats were housed in large metallic boxes with wire mesh lids under controlled temperature (20 °C–22 °C) and relative humidity (40%–50%) with 12 h light/darkness cycles and access to food and water *ad libitum*. All procedures were carried out in compliance with the ARRIVE guidelines and were approved by the Institutional Animal Care and Use Committee of the School of Medical Sciences, Universidad Nacional de Cuyo (Protocol approval No. 248/2024). Animals were culled at the end of experiments using an overdose of anesthetic (80 mg/kg ketamine plus 10 mg/kg xylazine). All behavioral observations and pharmacological interventions were carried out between 9 and 12 a.m.

We used female rats in this study for a number of reasons. First, most pain research has been conducted in male rats (around 90%) albeit the incidence of chronic pain is much higher in women than in men (Steingrimsdottir et al., 2017). Second, according to the CSBV (Considering Sex as a Biological Variable) provision introduced by the NIH in 2016 (Shansky and Murphy, 2021), preclinical studies must prioritize the population that exhibits the highest incidence of the pathologies in question. Third, a recent meta-analysis (Becker et al., 2016) has strongly refuted the myth that female responses in behavioral assays are more variable. In addition, studies have shown that the effect of the estrous cycle is rather limited in reflex and spontaneous behaviors (Levy et al., 2023). Finally, duplicating this very large study in males would have been extremely expensive and labor intensive, and against the guidelines of the 3R’s policy.

The total number of adult rats used for all experiments included in this work was 51 rats (not including the post-natal rats used for cell culture, see Section 2.11). Initially, we assessed the change in mRNA levels 14 days after chronic-contriction injury of the sciatic nerve (CCI). To achieve this, we used groups of 4 rats (normal or untreated, and three groups that received implantation of an ALZET minipump filled with either telmisartan or PD123319 or the combination of both drugs, see below for details). This amounted to a total of 16 rats. In the next phase of the study, 25 rats were used for tissue collection and immunostaining. These were divided as follows: 5 untreated, normal rats; 5 sham-operated; and then 5 for each time point we examined after CCI (3, 7 and 14 days). Finally, we conducted an experiment to assess the effectiveness of the treatments and to harvest tissue for immunostaining at 14 days after CCI. This amounted to a total of 20 additional rats. In summary, we used 9 rats per treatment in the behavioral assessment assays (the 4 per group coming from the qPCR assay plus the 5 per group used in the immunostaining). We took 5 blood samples at day 14 after CCI to determine plasma cytokine levels (by ELISA). This information has been summarized in the diagram of Supplementary Figure S1A.

### 2.2 Rat model of neuropathic pain

The CCI rat model was generated under deep anesthesia (50 mg/kg ketamine plus 5 mg/kg xylazine i.p.) following a recently improved procedure (Medeiros et al., 2021). The sciatic nerves on the left side were exposed, and then a double ligature was tied loosely around the nerve with approximately 1–2 mm space between knots. Rats in the sham group were subjected to sciatic nerve exposure without ligation. This procedure induces the whole range of signs and symptoms commonly seen with the standard 4-ligature model introduced in 1988 by Bennett, but shorten the operation time and improve recovery.

### 2.3 *In vivo* pharmacological studies

To study the possible role of the RAS system and TWIK1 in spontaneous pain, mechanical and cold-allodynia after CCI, we used subcutaneously implanted osmotic mini-pumps (model 2004, ALZET) filled with either a drug or vehicle. This choice of via of administration rests on four main reasons: one, it is not painful in itself for the rats; two, it is minimally invasive and does not require daily injections or excessive manipulation; three, delivers a known and calibrated dose of the drugs over a specified period of time and four, we have used this approach previously with success in models of neuropathic and inflammatory pain (Messina et al., 2022; Messina et al., 2024).

We used simple randomization (random.org/sequences) to assign the rats to each treatment. Each animal was identified utilizing a simple metallic tag. The control group received a mini-pump filled with sterile saline. All pumps were pre-filled with approximately 200 µL of the corresponding solutions and incubated for 40 h at 36 °C before implantation, as recommended by the manufacturer. Pump implantation into the humpback was performed simultaneously with the CCI surgery under deep anesthesia. Both, the investigator administering the drugs and the one evaluating behavior, were blinded as to the contents of the pump. Final data analysis was also performed single-blindly. For details of the behavioral tests see Section 2.4.

#### 2.3.1 AT1R inhibition by telmisartan

We used Telmisartan, a potent, long-lasting, nonpeptide antagonist of AT1 receptor. This compound exhibits analgesic activity in several models of pain. Telmisartan dissociates very slowly from the receptor with a dissociation half-life (t_1/2_) of 5.9 h and a longer terminal elimination half-life than other commercially available sartans (∼24 h) (Michel et al., 2013). The effective concentration for this compound ranges from 2 nM to 8 µM with a *K*i of 3.4 nM in rat lung and 14.4 nM for renal AT1R, with an average of 9.2 nM (Wienen et al., 2000). We chose a dose of 25 μg/kg/day equivalent to ∼1.2 µM in plasma. This concentration has been estimated to be below the range of effective hypotension while retaining significant binding to AT1R (Maillard et al., 2002). Telmisartan is a lipophilic compound with a partition coefficient log P = 3.2 (n-octanol/buffer at pH 7.4). Due to its physicochemical properties, this compound exhibits excellent tissue penetration (VD = 500 L), but it also renders it poorly soluble in aqueous solutions. For this reason, we used SACTAN® 40, from CASASCO labs, that provides individual pills each containing 40 mg of telmisartan in an excipient consistent of meglumine (12.5 mg), microcrystalline cellulose (128.975 mg), povidone K30 (3.75 mg), mannitol (37.5 mg), sodium starch glycolate (8 mg), sodium crosscarmellose (12 mg), sodium hydroxide (3.525 mg) and magnesium stearate (3.75 mg). These excipients greatly enhance solubility. Each tablet was finely pulverized to increase surface area and improve solubilization. The resulting powder was initially dissolved in 100% DMSO to prepare a concentrated stock solution within established solubility limits. Finally, this stock was diluted in saline to yield a final DMSO concentration of 2%, with gentle heating (42 °C for 10 min) and pH adjustment to 9.5 to further promote complete dissolution. This pH was chosen because the solubility of telmisartan in aqueous solutions is strongly pH-dependent, with maximum solubility observed at high (>9) and low (<3) pH (Wienen et al., 2000).

#### 2.3.2 AT2R inhibition by PD123319

To antagonize AT2R we used PD123319 di(trifluoroacetate) salt hydrate (Cat #P-245, Alomone labs, Israel), which given its formulation has a solubility of up to 100 mM in water. The effective concentration for this compound ranges from 1 nM to 10 µM with an IC50 of 5.09 nM in *in vitro* preparations (Toney and Porter, 1993). The drug also demonstrates potent activity with IC50 values of 34 nM in rat adrenal tissue and 210 nM in rat brain (Blankley et al., 1991). We chose a dose of 20 μg/kg/day equivalent to ∼1 µM in plasma, which is expected to be well within the range of effective concentrations for the receptor. This is lower than the dosage used in other studies aimed at examining the vascular and renal role of AT2R, which rely on acute i.v. administration and a short follow up. It is also important to note that subcutaneous, continuous release usually leads to plasma accumulation of the drug over 14 days. The drug was suspended in 2% DMSO diluted with saline. For the combination treatment we added the same doses of each antagonist, dissolved in the same vehicle.

#### 2.3.3 Reliability of drug delivery

To verify how efficiently the ALZET minipumps delivered the drugs, we followed the manufacturer’s recommendations. This involved evaluating residual reservoir volume after explantation of the pumps. Specifically, remaining fluid was aspirated using a blunt-end filling tube connected to a Hamilton microsyringe. In all cases, less than 5 µL of fluid remained, indicating near-complete delivery. In addition, *post hoc* visual inspection of the pumps revealed no evidence of precipitation, crystallization, or obstruction.

### 2.4 Behavioral tests

#### 2.4.1 Acclimation

We obtained a behavioral baseline for each rat, starting 3 days before the date of the CCI and osmotic pump implantation. We examined the behavioral responses at 3, 7, 11 and 14 days after CCI.

All behavioral tests were carried out during the light cycle between 9 and 12 a.m. Habituation was carried out by taking the rats once a day over 3 consecutive days to the testing room in their cage for 15 min each time. On the day of the testing, the animals were placed in an acrylic box (20 cm × 20 cm × 20 cm) with a removable acrylic floor for 5 min to measure Spontaneous Foot Lifting (SFL). Then, the acrylic floor was removed and, after 5 min, we performed the von Frey test to assess mechanical sensitivity. Finally, after an interval of 10 min, we performed the acetone (Choi’s) test. This order was the same on any given day and every week. Notice that no discernible impairment in mobility or motor control was observed in the animals used in this study.

#### 2.4.2 Spontaneous foot lifting test

Once the rat had become stationary, finished exploring, repositioning or grooming, we measured the cumulative number of SFL events during 5 minutes in the paws corresponding to the operated (from now on, the ipsilateral side) as well as the untreated side (contralateral). SFL is an accepted measurement of spontaneous pain (Djouhri et al., 2006).

#### 2.4.3 Assessment of mechanical sensitivity

Mechanical stimulation with von Frey filaments (Touch Test®, North Coast Medical Inc.) was applied in ascending and descending orders to the ipsilateral and contralateral hind paws. Each von Frey filament was applied 5 times at intervals of 3–5 s. The pattern of responses (either escape or no escape) was recorded and the paw mechanical withdrawal threshold (PWT) was estimated according to Chaplan’s protocol (Chaplan et al., 1994).

#### 2.4.4 Assessment of cold sensitivity

We observed the responses to a non-noxious cold stimulus by measuring the brisk foot withdrawal to the application of 100 μL of acetone (at room temperature) to the center of the plantar surface of both the contralateral and ipsilateral hind paw using a pipette. Nocifensive behaviors were scored from 0 to 3 (0 corresponds to no response; 1, a single withdrawal of the paw; 2, repetitive withdrawal of the paw and 3, scratching and/or licking of the affected hind paw and/or ankle) during 2 minutes immediately following either the acetone or warm water application following validated protocols (Choi et al., 1994).

### 2.5 Quantitative PCR (qPCR)

mRNA levels from either whole L4/L5 DRG or cultures with or without the corresponding treatments were determined by qPCR as previously described (Marsh et al., 2012; Messina et al., 2024). mRNA was extracted using RNeasy (Cat# 74104, Qiagen, Valencia, CA, United States). Total mRNA was quantified using a Nanodrop and only samples with an A260:A280 ratio above 1.8 were used. cDNA was synthesized from 200 ng total RNA using the M-MLV kit (Invitrogen®, cat. # 10338842, Thermo Fisher) supplemented with RNAseOut (Cat# 1077719, Thermo Fisher). TWIK1 and GAPDH mRNA were amplified using SsoAdvanced Universal SYBR Green Supermix (Bio-Rad, Cat# 1725271). Sequences are as follow: TWIK1 forward 5′-TGG​AGG​CCA​GCA​ATT​ATG​GA-3′; TWIK1 reverse 5′-GTG​GCC​ATA​GCC​TGT​GGT​G-3′; GAPDH forward 5′-CGC​ATC​TTC​TTG​TGC​AGT-3′; GAPDH reverse 5′-AAT​GAA​GGG​GTC​GTT​GAT​GG-3′. The qPCR program consisted of 2 min at 95 °C, 40 cycles of 15 s at 94 °C, 60 s at 60 °C, and 30 s at 72 °C, followed by one cycle of 30 s at 95 °C; 30 s a 50 °C and 30 s at 99 °C (Agilent Technologies, AriaMx Real-Time PCR System, Modelo: Serial No. MY18035224). GAPDH mRNA was used as loading control. We performed a negative control containing RNA instead of cDNA to rule out genomic DNA contamination. The primers were custom-designed in-house and checked for specificity using Primer-BLAST. The data were analyzed using the ΔΔCt method.

### 2.6 Western blotting (WB)

WB were performed to characterize the primary antibodies used in this study, as previously described (Acosta et al., 2014; Benitez et al., 2020). Tissue samples (L5 DRGs, cerebral cortex, cerebellum, thalamus and spinal cord) were extracted using Laemmli buffer supplemented with a protease/phosphatase inhibitor cocktail (HALT, Cat# 87786, Thermofisher, Waltham, MA, USA). Samples of 10 μg of total protein were run on 8%–10% polyacrylamide gels and transferred to PVDF membranes (Cat# GE10600023, Amersham Pharmacia Biotech, Piscataway, NJ, United States) before blotting. Membranes were blocked with 5% semi-skimmed milk dissolved in tris-buffer saline (TBS) during 1 h at RT and then probed overnight with 1:2000 rabbit anti-AT1R (sc-1173, RRID:AB_2305402) and 1:2000 rabbit anti-AT2R (NBP1-77368, RRID:AB_11018151). We used 1:2000 mouse anti-β-Tub III as loading controls. The membranes were washed and then blocked again for 30 min before incubation for 2 h at RT with either 1:2000 peroxidase-labeled anti-mouse (Cat# PI-2000-1, RRID:AB_2336198) or anti-rabbit (Cat# PI-1000, RRID:AB_2916034) from Vector Laboratories. The membranes were washed and the protein bands were developed using ECLPlus (Amersham) and visualized with the LAS-4000 System (Fujifilm).

### 2.7 Tissue preparation

After treatment, rats were anesthetized i.p. with 50 mg/kg ketamine (Cat#01750020010) plus 10 mg/kg xylazine (Cat# 01750020170), both from Richmond VetPharma and transcardially perfused with saline supplemented with heparin (10 UI/mL, Sigma, Cat# H3393) followed by Zamboni’s fixative (Stefanini et al., 1967). Next L4/L5 DRGs were dissected and post-fixed for 1 h in Zamboni’s at RT and dipped in sequentially through graded sucrose solutions (from 10% to 30%) at 4 °C. Tissue was frozen and kept at −70 °C until used. Serial 10 μm cryostat sections were cut and placed on gelatin-coated slides before immunostaining.

### 2.8 Immunohistochemistry and antibody characterization

Normal, untreated L4/L5 DRG from 5 rats were used to conduct the initial characterization of TWIK1 staining. To achieve this goal, we used ABC/DAB labeling, using previously described protocols (Acosta et al., 2014; Benitez et al., 2017; Haskins et al., 2017).

For double immunofluorescence staining of L4/L5 DRG sections from 5 experimental rats per group, mid-cryosections of 10 μm were rehydrated with PBS for 10 min and then permeabilized 5 min with 0.2% v/v triton-X100 and blocked for 3 h at RT with 5% BSA. Goat anti-TWIK1 (1:400) staining was performed overnight at 4 °C followed by rinsing with PBS and incubation 2 h at room temperature with a donkey anti-goat Alexa 488 or horse anti-goat Alexa 594 (1:500). After rinsing of the secondary antibody with PBS, we used counter-staining with Isolectin B4 (IB4), which labels small, C-nociceptor neurons in the DRG (Fang et al., 2006) or trk-A (rabbit anti-trkA 1:2000, cat. # 06-574, MD Millipore Corp.) which labels primarily peptidergic neurons (Fang et al., 2005). DAPI (1:1000, 30 min incubation) was used in some instances. We followed the same procedure as with TWIK1 to perform staining against AT1R using 1:1000 rabbit anti-AT1 antibody from Santa Cruz (Cat. # sc-1173) or against AT2R with 1:2000 rabbit-anti AT2 from Novus Biologicals (Cat. # NBP1-77368). All tissues were given a final wash and then mounted with FluorSave. We employed staining against GFAP to evaluate satellite-glial cells reactivity. The tissue sections were treated as above and incubated overnight with a mouse anti-GFAP (1:400, clone 8-1E7-s, obtained from DSHB). A donkey anti-mouse conjugated with Alexa 594 (1:500) was used as secondary, combined with counterstaining using DAPI (1:1000).

The goat anti-TWIK1 (H-20, sc-11483, RRID:AB_2130786) has already been used and characterized in the literature (Deng et al., 2007; Deng et al., 2009). Furthermore, we have recently performed a full characterization of this antibody, which can be found in the supplementary data of that paper (Peralta et al., 2026).

Antibodies against AT1R (sc-1173, RRID:AB_2305402) and AT2R (NBP1-77368, RRID:AB_11018151) were characterized by Western blot (WB) and immunohistochemistry. Homogenates from the central nervous system (cortex, cerebellum, thalamus, and spinal cord) and the peripheral nervous system of adult rats were analyzed by WB. As shown in Supplementary Figure S2, single bands of varying intensity were detected across the different tissues, at the expected molecular weights reported in the literature for AT1R and AT2R (Yu et al., 2010).

To further establish antibody specificity by immunohistochemistry, rat kidney tissue was used because its expression pattern is well characterized in the literature. Miyata et al. reported the localization of AT1R and AT2R throughout the rat kidney, with the highest staining intensity observed in the vasculature of the renal cortex and in the proximal tubules of the outer medulla. In contrast, AT2R expression was scarce or absent in the glomeruli and in the thick ascending limb of the loop of Henle (Miyata et al., 1999). Both antibodies used in this study showed an expression pattern similar to that previously described in renal tissue, mainly labeling the renal tubules (white arrow, Supplementary Figure S2) and, to a lesser extent, the renal glomeruli (yellow arrow, Supplementary Figure S2).

### 2.9 Image acquisition and analysis

The quantitative analysis of immunostaining was performed on images captured with an Olympus FV-1000 confocal or a Zeiss Axio Observer microscopes using well established and previously validated and detailed procedures (Benitez et al., 2017; Benitez et al., 2020; Messina et al., 2022). The quantification was carried out using the software HCImage (Hamamatsu, RRID:SCR_015041). For each comparison, four to six fields were captured at ×40 magnification with identical settings during the same session. All intensity measurements were performed at the neuronal soma and only neurons with clearly visible nuclei were included in the analysis. Both subjective and objective approaches were used for image analysis. For the subjective scoring cytoplasmic immunoreactivity was graded as follows: 0 = negative; 1 = just visible above background; 5 = strongest observed staining within the section, with intermediate values assigned according to perceived intensity.

For objective quantification, cytoplasmic mean pixel density (c) was measured for each neuron. To normalize staining intensity across sections, the mean pixel densities of the five least intensely stained neurons (a, representing background level; 0%) and the five most intensely stained neurons (b, representing maximal signal; 100%) were also determined. The percentage relative intensity was then calculated as (100 × ((c − a)/(b − a))%).

To define the threshold for positivity, subjective scores were plotted against the corresponding normalized intensity values after completion of the full analysis. This relationship was used to determine the cut-off corresponding to background staining, which was subsequently applied to distinguish negative from positive neurons. This procedure and the resulting threshold definition are illustrated in Supplementary Figure S3.

Neurons were subdivided according to profile size as previously described: lumbar DRG neuron sizes (cross-sectional areas) in this age and gender of rats are related to fiber CV as follows: small (up to 400 μm^2^) mostly have C-fibers, medium-sized neurons (400–800 μm^2^) mostly have Aδ-fibers and large neurons (>800 μm^2^, including only the upper part of the large neuron distribution) have Aα/β fibers.

The ImageJ software was used for quantification of GFAP. Confocal images were converted to 8-bit format. For each image, random 100 μm^2^ areas were selected from tissue regions containing neurons. A threshold was applied to identify GFAP-immunoreactive signal, and percentage of marked area by the anti-GFAP antibody within each sampled region was measured. The GFAP-positive area was expressed as a percentage of the 100 μm^2^ region for each image, and results were plotted as a scatter bar graph.

### 2.10 Enzyme-linked immunosorbent assay (ELISA)

#### 2.10.1 Plasma collection from rat blood

At day 14 after CCI, approximately 3 mL of blood was drawn from 5 rats per experimental group by heart puncture and collected in heparin-containing vials. These samples were incubated for 60 min at 37 °C, then kept overnight at 4 °C. The next day, the samples were centrifuged in a cooling centrifuge at 10,000 rpm for 10 min, and clear non-hemolyzed plasma samples were obtained, transferred to fresh microfuge tubes and preserved at −80 °C, until further assessment.

#### 2.10.2 Quantification of plasma cytokines

We measured four cytokines in plasma using the following kits: Rat IL-6 DuoSet ELISA (Cat # DY506), Rat IL-10 DuoSet ELISA (Cat # DY522), Rat IL-1 beta/IL-1F2 DuoSet ELISA (Cat # DY501), Rat TNF-alpha DuoSet ELISA (Cat # DY510) and DuoSet® Ancillary Reagent Kit 2 (Cat # DY008). All kits were from R&D Systems (a Biotechne Brand). The manufacturer’s protocols were followed, including the construction of calibration curves using purified cytokines as standards. Absorbance values at 450 nm and 570 nm (as the reference wavelength) were measured using a VANTAstar (BMG LabTech).

### 2.11 Cell cultures

To examine the effect of pro-inflammatory cytokines on the mRNA levels of TWIK1, DRGs from postnatal day 6 (P6) rats (one per experimental set; a single culture derived from 1 rat was considered as an independent repetition), were dissected out following previously detailed protocols (Messina et al., 2022). This age was chosen because the neurons attached very well to collagen, exhibit high viability and contains a representative set of phenotypes compared to adult DRG neurons. Briefly, the ganglia were enzymatically digested at 37 °C in 0.25% w/v trypsin (Gibco, Cat. # 27250018) for 25 min in HBSS without divalent cations, washed and then incubated with 0.5% type I collagenase (Gibco, Cat. # 17100017) in HBSS for 20 min. The enzymes were subsequently quenched with DMEM/F12 (Gibco, Cat. # 11320033) supplemented with 5% fetal bovine serum (Natocor, Argentina), centrifuged and then mechanically dissociated. Isolated cells were plated directly onto Petri dishes filled with 2 mL of DMEM/F12 supplemented with B-27 (Gibco, Cat. # 17504-044) and penicillin/streptomycin (Gibco, Cat. # 15140122) and coated with rat tail collagen (300 ng/mm^2^). Neurons were allowed to attach to the substratum for 3–4 h before addition of the cytokines. Cells were kept for 2 or 3 days *in vitro* (DIV) at 37 °C in a 5% CO_2_ incubator. Cytokines were freshly added every 24 h. Initial neuron density was standardized to ∼10,000 cells/mL.

The control group received no treatment, and the treated group received an inflammatory soup (IS) containing: 100 nM IL-6 (Cat. #I0406), 1 μM TNF-α (Cat. #T5944), and 1 µM IL-1β (Cat. #I2393). All drugs obtained from Sigma.

### 2.12 Statistical analysis

All tests were performed with Prism 7 (GraphPad software RRID:SCR_000306). Descriptive results are shown as means ± SEM. The normal distribution of the data was assessed with the D’Agostino-Pearson test. All tests performed were two-tailed and a level of p < 0.05 was considered statistically significant. Significance is indicated on all graphs by *p < 0.05, **p < 0.01, ***p < 0.001, ****p < 0.0001. Magnitude of the effects was taken into account for the interpretation of the data sets, as statistical significance alone might mask them. Behavioral data were analyzed using two-way repeated-measures ANOVA, followed by Dunnett’s multiple comparisons test for specific day and treatment where appropriate. For other types of data, comparisons among multiple groups were performed by Kruskall-Wallis one-way ANOVA plus Dunn’s test. Differences between two groups (typically contralateral vs. ipsilateral for a given time or treatment) were evaluated using the Mann-Whitney U test.

## 3 Results

### 3.1 Characterization of TWIK1 expression in adult rat DRG neurons

**FIGURE 1:**
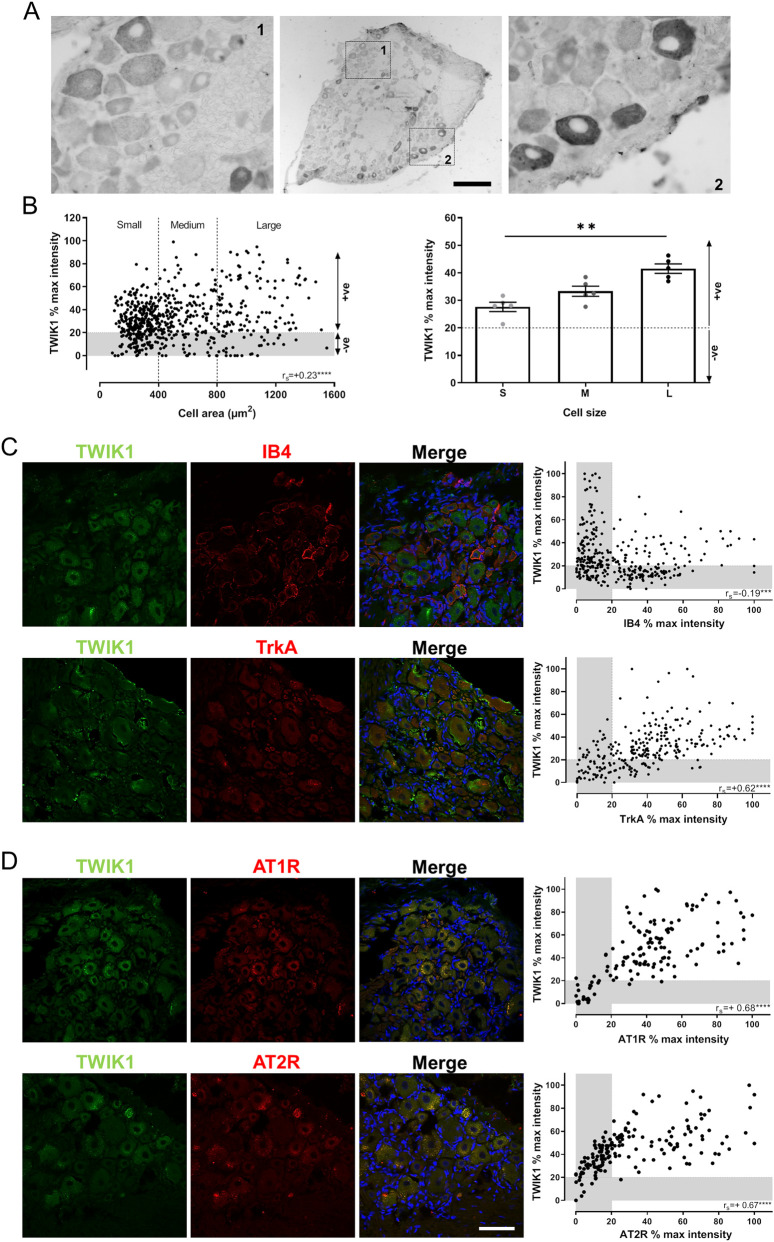
TWIK1 and AT1R/AT2R expression in DRG neurons. **(A)** Representative ABC/DAB images of TWIK1 expression in whole L4 DRG. Insets show higher magnification views (×40). Scale bar: 250 μm; inset: 50 µm. **(B)** Quantification of TWIK1 staining. Left: correlation between neuronal cell area and mean cytoplasmic TWIK1 intensity (Spearman’s rs = 0.23, *p* < 0.0001). Right: percentage of cytoplasmic TWIK1 intensity by neuronal size (small, medium, large). Data are presented as mean ± SEM. **(C)** Representative immunofluorescence images showing co-localization of TWIK1 with IB4 (non-peptidergic neurons) and TrkA (peptidergic neurons). Correlation analysis shows a negative association with IB4 (rs = −0.19, *p* = 0.0004) and a positive association with TrkA (rs = 0.62, *p* < 0.0001). **(D)** Representative immunofluorescence images showing co-localization of TWIK1 with AT1R and AT2R in L5 DRG. Positive correlations were observed for both receptors (rs = 0.68 and 0.67, respectively; *p* < 0.0001). Scale bar: 50 µm.

Figure 1A shows the pattern of TWIK1 expression in whole adult L4 DRG as revealed by ABC/DAB staining. Insets (labeled 1 and 2) correspond to images captured at ×40 magnification and provide a detailed view of channel expression and intensity. Quantitative immunohistochemistry demonstrated a statistically significant positive correlation (Spearman’s r_s_ = +0.23, p < 0.0001) between the average cytoplasmic % intensity of TWIK1 and neuronal cell area (Figure 1B, left panel). The mean ± SEM values of TWIK1 percentage intensity for neuronal subpopulations classified by size were: small 27.6% ± 1.7%, medium 33.3% ± 1.8%, and large 41.5% ± 1.7%. A significantly higher intensity was observed in large compared to small neurons (p = 0.0044, Kruskal–Wallis statistic = 10.26, Figure 1B, right panel).

We next examined which phenotypes of DRG neurons expressed TWIK1. Figure 1C shows representative images of co-localization between TWIK1 and IB4 (top panel) and trkA (bottom panel), indicative of non-peptidergic and peptidergic sensory neurons, respectively. Co-localization with IB4-binding neurons showed a negative Spearman correlation (r_s_ = −0.19, p = 0.0004, n = 340 neurons), whereas TWIK1 exhibited a positive correlation with trkA (r_s_ = +0.62, p < 0.0001, n = 275 neurons).

Given the focus on Ang-II receptor signaling, we assessed whether TWIK1 co-localized with AT1R and AT2R. Figure 1D displays representative photomicrographs of TWIK1 staining with AT1R (top panel) or AT2R (bottom panel) in normal L5 DRG. Significant positive Spearman correlations were observed between TWIK1 and both receptors (r_s_ = +0.68 and r_s_ = +0.67 for AT1R and AT2R, respectively; both p < 0.0001).

### 3.2 TWIK1 expression decreases in the L5 DRG after CCI and is modulated by pharmacological inhibition of AT1R and/or AT2R

We first confirmed that the CCI procedure resulted in damage to DRG neurons using ATF-3 as a marker. Supplementary Figure S4A shows representative ABC/DAB images of L5 DRG obtained 3 days after sham surgery or CCI (contralateral and ipsilateral). ATF-3 nuclear staining was evident on the ipsilateral side.

**FIGURE 2:**
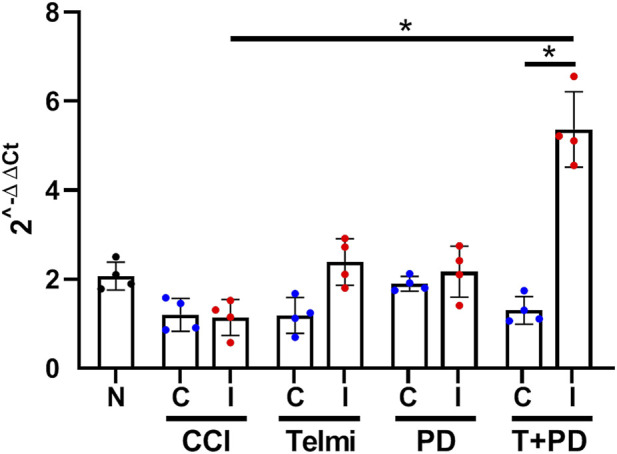
TWIK1 mRNA expression in L4/L5 DRG after CCI and angiotensin receptor blockade. Female Wistar rats were treated with telmisartan, PD123319, or their combination (Telmi + PD). TWIK1 mRNA levels were normalized to GAPDH. Data are presented as mean ± SEM (N = 4 per group). TWIK1 expression was increased in the ipsilateral Telmi + PD group compared with the contralateral side (*p* = 0.0284) and ipsilateral CCI (*p* = 0.0174). Statistical analysis: Kruskal–Wallis with Dunn’s *post hoc* test.

A pilot study (N = 4 rats per group) was conducted to evaluate behavioral responses (see Section 2.4) and TWIK1 transcript levels. Following 14 days of treatment with telmisartan, PD123319, or their combination (Telmi + PD), RNA was extracted from L4–L5 DRG and RT-qPCR was performed. TWIK1 levels relative to GAPDH were increased in the ipsilateral T + PD group compared to its contralateral side (p = 0.0284) and ipsilateral CCI (p = 0.0174, KW = 27.99) (Figure 2).

We next examined TWIK1 protein expression. No significant differences were observed between normal and sham-operated rats (Supplementary Figure S4B). Mean ± SEM percentage intensities were: small neurons 28.1 ± 0.7 vs. 35.7 ± 3.5 (p = 0.0952, U = 4); medium neurons 32.9 ± 2.0 vs. 37.5 ± 2.7 (p = 0.2222, U = 6); large neurons 39.8 ± 1.9 vs. 34.1 ± 1.5 (p = 0.0952, U = 4).

**FIGURE 3:**
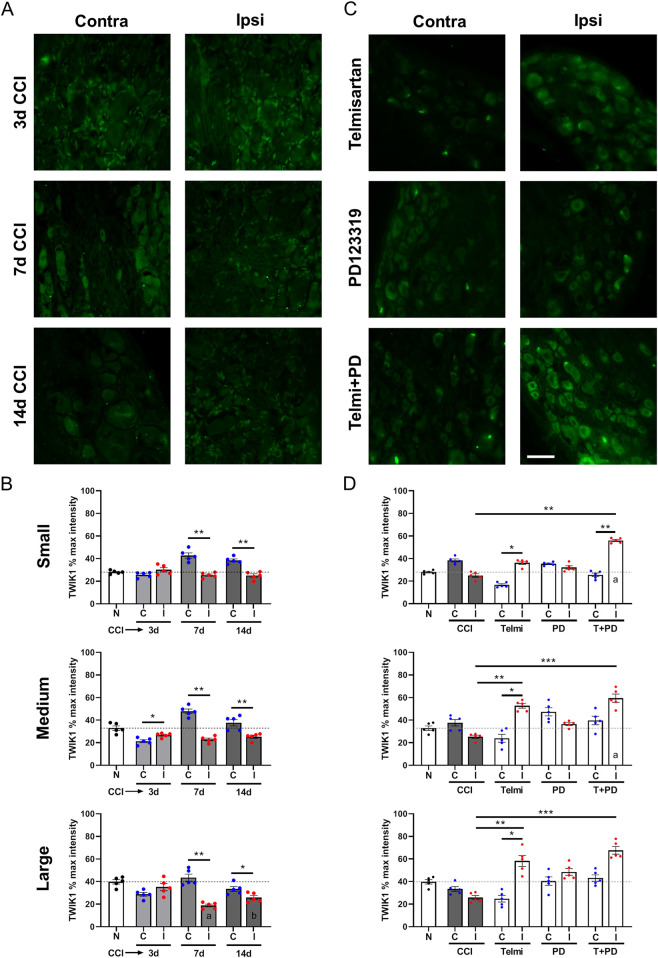
TWIK1 expression in DRG neurons after CCI and pharmacological modulation of angiotensin II receptors. **(A)** Representative immunofluorescence images of TWIK1 in L4/L5 DRG from contralateral and ipsilateral sides at 3, 7, and 14 days after CCI. **(B)** Quantification of TWIK1 staining by neuronal size (small, medium, large) at each time point. **(C)** Representative images of TWIK1 staining after 14 days of treatment with telmisartan, PD123319, or their combination (T + PD). **(D)** Quantification of TWIK1 staining expressed as percentage of maximum cytoplasmic intensity. Data are presented as mean ± SEM (N = 5 per group). Statistical analysis: Kruskal–Wallis with Dunn’s *post hoc* test; contralateral vs. ipsilateral comparisons by Mann–Whitney U test. Scale bar: 50 µm.

Figures 3A,C show representative images of TWIK1 staining in L5 DRG sections from contralateral and ipsilateral sides at 3, 7, and 14 days after CCI, and after 14 days of pharmacological treatment. Quantitative analysis (Figure 3B) was performed in 5 rats per time point.

In small neurons, no differences were observed at 3 days CCI (25.7% ± 1.1% vs. 30.1% ± 1.9%, p = 0.1667, U = 5), whereas significant differences between contralateral and ipsilateral sides were observed at 7 days (25.4% ± 1.0% vs. 42.7% ± 2.4%, p = 0.0079, U = 0) and 14 days (25.0% ± 1.5% vs. 38.4% ± 1.3%, p = 0.0079, U = 0). Notice that these ipsilateral levels are similar to the values present in normal rats.

In medium-sized neurons, no significant differences were observed relative to normal. However, comparisons between contralateral and ipsilateral sides revealed differences at 3 days (20.7% ± 1.6% vs. 26.7% ± 0.9%, p = 0.0159, U = 1), 7 days (47.8% ± 2.0% vs. 22.9% ± 1.2%, p = 0.0079, U = 0), and 14 days (37.7% ± 2.8% vs. 25.3% ± 1.4%, p = 0.0079, U = 0).

In large neurons, TWIK1 intensity was reduced ipsilaterally at 7 and 14 days compared to normal (18.8% ± 1.2% and 25.8% ± 1.8%, p = 0.0011 and p = 0.0473, respectively). Significant differences between contralateral and ipsilateral sides were observed at 7 days (43.5% ± 3.2% vs. 18.8% ± 1.2%, p = 0.0079, U = 0) and 14 days (34.4% ± 2.2% vs. 25.8% ± 1.8%, p = 0.0492, U = 1).

Our question at this stage in the research is: does TWIK1 channel expression changes as a result of the pharmacological modulation of the Ang-II receptors? And if so, in what direction does this regulation move? We assessed these topics in the following sections of the manuscript.

We next assessed the effects of pharmacological inhibition of Ang-II receptors (Figures 3C,D). In small neurons, the combination treatment (T + PD) increased TWIK1 expression ipsilaterally compared to normal (56.1 ± 1.0 vs. 28.1 ± 0.7, p = 0.0381, KW = 39.94) and CCI 14d ipsilateral (56.1 ± 1.0 vs. 25.0 ± 1.5, p = 0.0046). A decrease was also observed contralaterally with telmisartan (p = 0.0120). T + PD ipsilateral values were higher than T + PD contralateral (p = 0.0091).

In medium neurons, T + PD increased TWIK1 compared to normal (59.5% ± 3.7% vs. 32.9% ± 1.9%, p = 0.0193). Telmisartan and T + PD increased TWIK1 relative to CCI 14d (p = 0.0065 and p = 0.0010, respectively). Telmisartan also increased ipsilateral TWIK1 (p = 0.0110, KW = 35.18).

In large neurons, telmisartan and T + PD increased TWIK1 compared to CCI 14d ipsilateral (58.2% ± 4.8% and 67.7% ± 3.3% vs. 25.8% ± 1.8%, p = 0.0100 and p = 0.0007, respectively). Telmisartan also increased TWIK1 expression (p = 0.0091, KW = 36.24).

The next step in our study consisted in a thorough evaluation of the behavioral impact of using a continuous administration of the inhibitors of AT1R, AT2R or both in the CCI model. We aimed at establishing whether these treatments that upregulate the expression of TWIK1 (reversing the effects of CCI alone) have an impact on chronic pain.

### 3.3 Selective *in vivo* antagonism of AT1R and AT2R alleviates neuropathic pain

**FIGURE 4:**
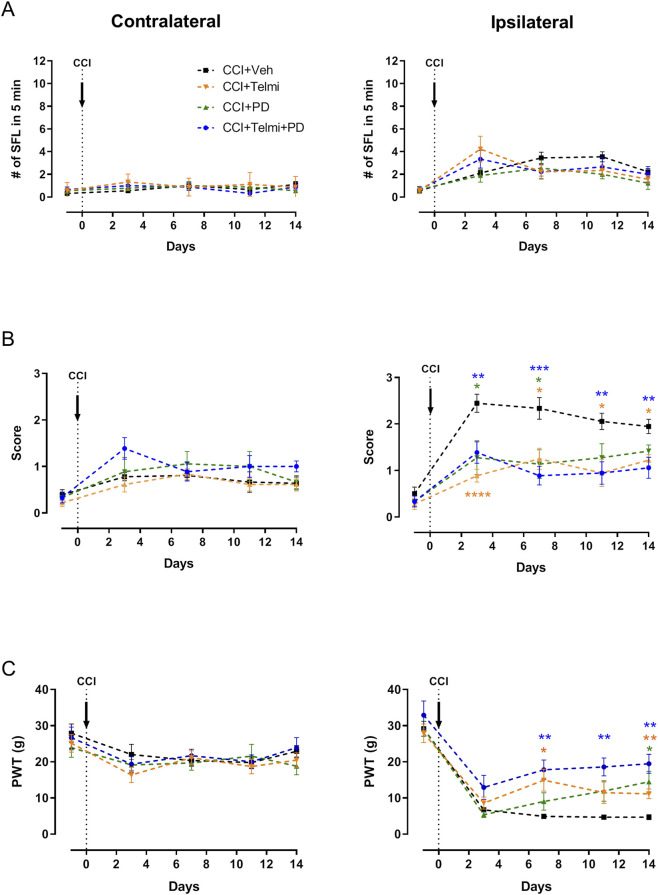
Effects of AT1R and AT2R antagonists on neuropathic pain behavior after CCI. Time-course analysis of behavioral responses in contralateral and ipsilateral hind paws (N = 9 per group). **(A)** Spontaneous foot lifting (SFL; events/5 min). **(B)** Cold allodynia assessed by Choi’s test (score 0–3). **(C)** Mechanical sensitivity measured as paw withdrawal threshold (PWT; von Frey test). Data are presented as mean ± SEM. Statistical analysis: two-way repeated-measures ANOVA followed by Dunnett’s *post hoc* test.

Figure 4 summarizes behavioral data from nine rats per group. Spontaneous foot lifting (SFL) showed no significant treatment effects contralaterally (F(3,32) = 0.4930, p = 0.6897) or ipsilaterally (F(3,32) = 0.4727, p = 0.7035) (Figure 4A). Albeit telmisartan appears to induce a slightly larger number of SFL events at day 3, this did not reach statistical significance relative to CCI-vehicle or any other condition.

Cold allodynia (Figure 4B) showed no differences contralaterally (F(3,32) = 1.671, p = 0.1928). Ipsilaterally, treatments reduced CCI-induced allodynia at 3 days (F(3,32) = 9.092, p = 0.0002): telmisartan (p < 0.0001), PD (p = 0.0383), and T + PD (p = 0.0086). Effects persisted at 7 days and partially at 11 and 14 days. At the end of the observational period, cold allodynia in the CCI-vehicle eased-off, and thence the treatments had a moderate effect.

Mechanical sensitivity (Figure 4C) was unaffected contralaterally (F(3,32) = 0.6039, p = 0.6173). Ipsilaterally, treatments were significant (F(3,32) = 10.39, p < 0.0001). The Dunnett’s multiple comparisons tests show that at 7 days Telmi (p = 0.0288) and Telmi + PD (p = 0.0038) alleviate mechanically-induced pain in a significant manner compared to CCI + vehicle. At 11 days, only the combination of Telmi + PD (p = 0.0012) remains effective. Finally, at CCI 14d, Telmi (p = 0.0032), PD (p = 0.0252) and Telmi + PD (p = 0.0011) all improved mechanically-induced pain.

Thus, we did not find evidence for a significant involvement of the RAS system in CCI-triggered spontaneous pain. However, both, cold allodynia and mechanical hypersensitivity appear to be alleviated by the continuous administration of selective inhibitors of AT1R and/or AT2R after the injury to the sciatic nerve.

### 3.4 CCI and pharmacological treatments alter AT1R and AT2R expression in DRG neurons

**FIGURE 5:**
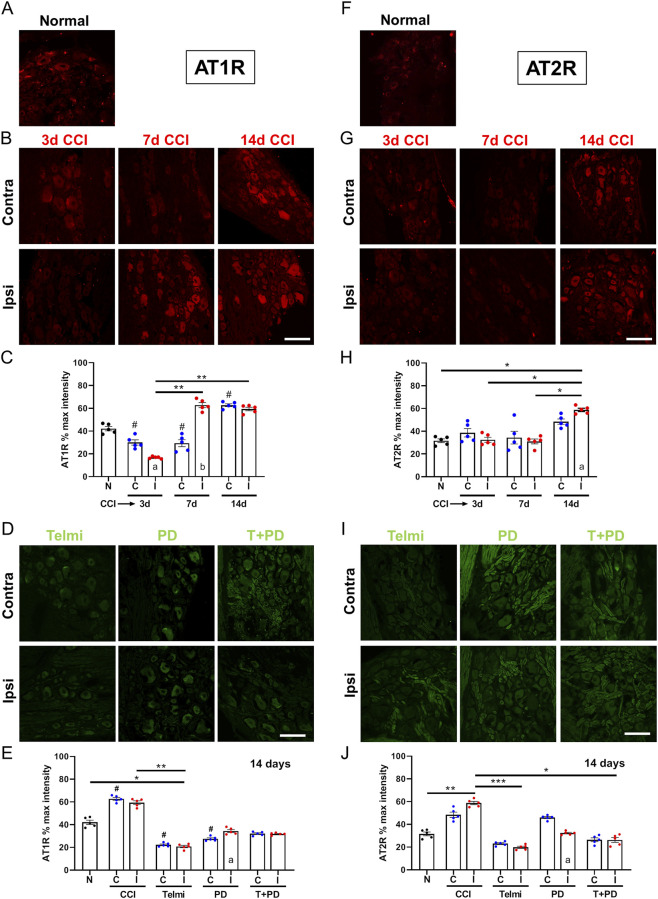
AT1R and AT2R expression in L4/L5 DRG neurons after CCI and angiotensin receptor modulation. **(A,B)** Representative confocal images of AT1R staining in normal, contralateral and ipsilateral DRG at 3, 7, and 14 days after CCI. **(C)** Quantification of AT1R staining at each time point. **(D)** Representative images after 14 days of treatment with telmisartan, PD123319, or T + PD. **(E)** Quantification of AT1R staining expressed as percentage of maximum cytoplasmic intensity. Data are presented as mean ± SEM (N = 5 per group). Statistical analysis: Kruskal–Wallis with Dunn’s *post hoc* test; Mann–Whitney U test for contralateral vs. ipsilateral comparisons. Scale bar: 50 µm. **(F,G)** Representative immunofluorescence images of AT2R staining in normal and also contralateral and ipsilateral DRG at 3, 7, and 14 days after CCI. **(H)** Quantification of AT2R staining at each time point. **(I)** Representative images after 14 days of treatment with angiotensin receptor antagonists. **(J)** Quantification of AT2R staining expressed as percentage of maximum cytoplasmic intensity. Data are presented as mean ± SEM (N = 5 per group). Statistical analysis: Kruskal–Wallis with Dunn’s *post hoc* test; Mann–Whitney U test for contralateral vs. ipsilateral comparisons. Scale bar: 50 µm.

AT1R expression (Figures 5A–C) decreased ipsilaterally at 3 days CCI (16.8% ± 0.4% vs. 42.1% ± 1.9%, p = 0.0159, KW = 30.38), increased at 7 days (62.8% ± 2.2%, p = 0.0016), and remained elevated at 14 days (59.4% ± 1.6%, p = 0.0081). Contralateral increases were observed at 14 days (p = 0.0473). Differences between sides were significant at 3 and 7 days (p = 0.0079). This suggests that in the long term there is a systemic component to the regulation of AT1R in the DRG in this model of neuropathic pain.

Figures 5D,E presents the images and data for AT1R cytoplasmic % intensity in the L5 DRG neurons after 14 days treatment with telmisartan, PD123319 and the combination. After treatment, AT1R levels were reduced, particularly with telmisartan (20.8% ± 1.1% vs. 42.1% ± 1.9%, p = 0.0226; vs. CCI 14d ipsilateral p = 0.0010, KW = 41.45). A significant ipsilateral–contralateral difference was observed only for PD (p = 0.0079).

Figures 5F,G include example images of normal, contralateral and ipsilateral images 3, 7 and 14 days after CCI of AT2R immunostaining (in red) or at 14 days (in green) in the presence of the different treatments (Figure 5I). The scatter bar plots summarize the results of the quantitative analysis of the IHC staining at each time point and experimental condition (Figures 5H,J).

AT2R expression showed no differences at 3 or 7 days but increased at 14 days CCI (58.8 ± 1.5 vs. ∼31–32%, p ≈ 0.02, KW = 20.69). A difference between sides was observed at 14 days with a slight increase ipsilaterally (58.8% ± 1.5% vs. 48.5% ± 2.4%, MW, p = 0.0159, U = 1).

Comparison between the treated sides showed a significant drop in the expression levels of AT2R between ipsilateral CCI 14d (58.8% ± 1.5%) and both, telmisartan (vs. 19.8% ± 1.0%, p = 0.0001) and the combination treatment (vs. 26.2% ± 2.1%, p = 0.0298, KW = 35.17). When comparing ipsi and contra for each treatment, we encountered a statistically significant reduction in AT2R only for PD123319 (32.3% ± 0.7% vs. 46.0% ± 1.0%, MW, p = 0.0079, U = 0). Overall, it seems that administration of selective antagonists of AT1R or AT2R result in a significant reduction in the levels of expression for both receptors after 14 days.

Taken together, our results demonstrate that the combination treatment is the most consistent in terms of alleviating neuropathic pain and up-regulating TWIK1 over time. Considering the prominent role described in the literature for AT1 and AT2 receptors in inflammation, it is possible that the actions of the negative modulators used in this study involve other mechanisms apart from the upregulation of TWIK1, most prominently inflammation secondary to the lesion of the sciatic nerve. To explore this possibility, we next determined the plasma levels of various cytokines.

### 3.5 Effects of treatment on circulating cytokines

**FIGURE 6:**
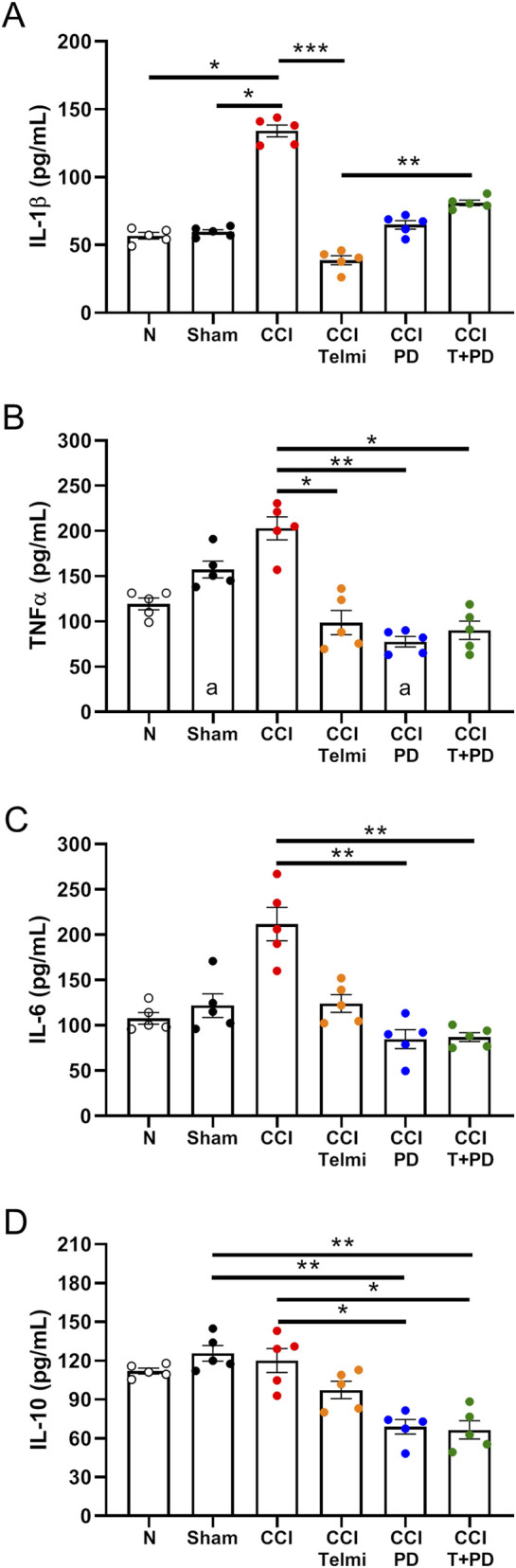
Circulating cytokine levels after CCI and angiotensin receptor antagonism. Plasma levels (pg/mL) of IL-1β **(A)**, TNF-α **(B)**, IL-6 **(C)**, and IL-10 **(D)** were measured by ELISA at 14 days after CCI. Experimental groups included control, sham, CCI, and treated animals (telmisartan, PD123319, T + PD). Data are presented as mean ± SEM. Statistical analysis: Kruskal–Wallis with Dunn’s *post hoc* test.

Figure 6 displays scatter bar plots showing the average ± SEM concentration (in pg/mL) of IL-1β (A), TNF-α (B), IL-6 (C) and IL-10 (D) determined by ELISA at 14 days after CCI. For IL-1β, we found a significant increment in CCI relative to normal (134.0 ± 4.4 vs. 56.8 ± 2.4 pg/mL, p = 0.0267, KW = 25.89) and sham (59.6 ± 1.6 pg/mL, p = 0.0430). Treatment with telmisartan resulted in a reduction to 38.7 ± 3.4 pg/mL (p = 0.0001), but the combination treatment caused a small but significant increment to 81.1 ± 2.0 pg/mL (p = 0.0049).

TNF-α levels tended to increase in the CCI rats, but this increase did not reach statistical significance when compared against normal (202.8 ± 12.7 vs. 119.4 ± 6.4 pg/mL). The three treatments (telmisartan, PD123319 and T + PD) caused a significant reduction in the plasmatic concentration of this pro-inflammatory cytokine: 98.7 ± 13.3, 77.7 ± 5.7 and 90.1 ± 10.1 pg/mL respectively, with p values of 0.0382, 0.0017 and 0.0133, KW = 23.27.

A similar picture emerged for IL-6; after 14d CCI, the levels reached 211.7 ± 18.4 against 107.7 ± 6.4 pg/mL in the normal rats. Despite almost duplicating the average value, this difference did not reach statistical significance. When compared to CCI, treatment with PD and T + PD resulted in substantial drops to 84.8 ± 10.4 and 86.9 ± 4.9 pg/mL with p = 0.0028 and 0.0016 respectively (KW = 21.53).

Finally, the anti-inflammatory cytokine IL-10 exhibited a distinct pattern where the levels did not increase after 14 days CCI, while remaining elevated in the sham rats, a finding that agrees with previous reports (Jancalek et al., 2010). Notwithstanding this, we found some significantly reduced levels of IL-10 for PD (68.9 ± 5.6 pg/mL) and T + PD (66.4 ± 7.1 pg/mL) compared to sham (125.7 ± 6.1 pg/mL, p = 0.0085 and 0.0097) and CCI (120.1 ± 9.2 pg/mL, p = 0.0430, KW = 21.52).

Thus, especially the combination treatment seems to be effective at lowering the levels of circulating pro-inflammatory cytokines, while IL-10 accompanies this process. This poses the question of whether and how do the treatments alter the reactivity of satellite glial cells within the affected DRGs. Furthermore, is this another complex link in a series of events leading to the consolidation of a chronic inflammatory (painful) state that cannot be reversed by our pharmacological interventions once established? We tackled this question in the next section.

### 3.6 Chronic negative regulation of AT1 and/or AT2 receptors in the CCI-operated rats alters the reactivity of satellite glial cells within the DRG

**FIGURE 7:**
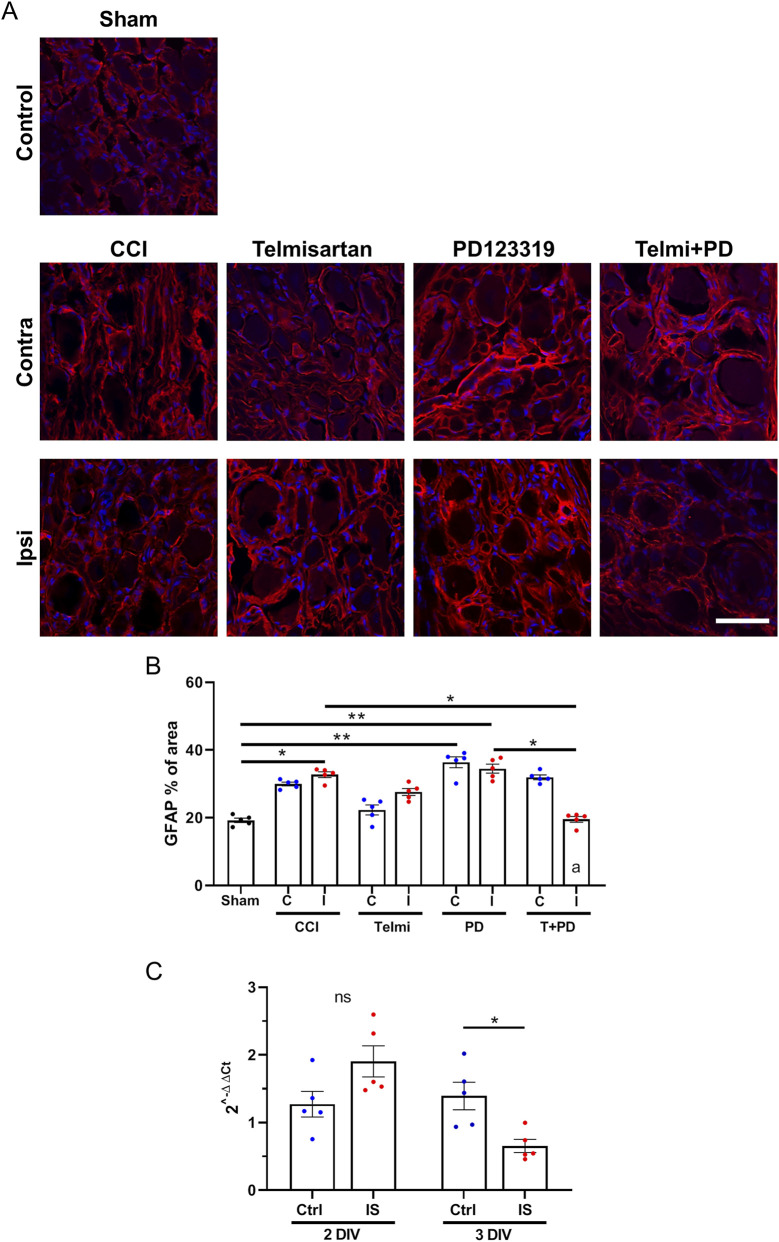
GFAP expression and TWIK1 mRNA levels after CCI and angiotensin receptor modulation. **(A)** Representative immunofluorescence images of GFAP staining in L5 DRG from contralateral and ipsilateral sides in sham, 14-day CCI, and treated animals. **(B)** Quantification of GFAP-positive area (% total area). Data are presented as mean ± SEM (N = 5 per group). Statistical analysis: Kruskal–Wallis with Dunn’s *post hoc* test; Mann–Whitney U test for pairwise comparisons. Scale bar: 40 µm. **(C)** TWIK1 mRNA levels in cultured DRG neurons treated for 2 or 3 days with inflammatory cytokines (IL-1β, IL-6, TNF-α; inflammatory soup, IS) or control (Ctrl), normalized to GAPDH. Data are presented as mean ± SEM.

Figure 7 presents the images (A) and data (B) corresponding to GFAP expression within L5 DRG sections in sham, 14d CCI and 14d CCI plus the treatments, both contralaterally and ipsilaterally. The analysis consisted in quantifying the % of total area occupied by GFAP + ve cells (primarily satellite glial cells) at each condition.

GFAP analysis (Figures 7A,B) showed increased ipsilateral area after CCI (32.7% ± 0.9% vs. 19.2% ± 0.7%, p = 0.0381). PD123319 did not reverse this increase. T + PD reduced GFAP + area ipsilaterally compared to CCI and PD123319 (p = 0.0472 and p = 0.0132, KW = 37.55). A significant ipsilateral reduction was observed only with T + PD (p = 0.0079, U = 0). This may reflect both systemic and local effects that translate into a more pronounced decline in the activation state of SGC.

### 3.7 Inflammatory modulation of TWIK1 mRNA levels in cultured DRG neurons

Finally, we tested the hypothesis that the potential main mechanism coupling activation of AT1R and AT2R by Ang-II (which itself goes up after sciatic nerve injury) is the release of pro-inflammatory cytokines that in turn reduces the expression of TWIK1 in primary sensory neurons.

Treatment with inflammatory soup did not significantly alter TWIK1 mRNA at 2 days (p = 0.0556, U = 3), but reduced expression at 3 days (p = 0.0317, U = 2) (Figure 7C).

## 4 Discussion

The present study provides evidence supporting a functional relationship between angiotensin II (Ang-II) receptors (AT1R and AT2R) and the K2P channel TWIK1 in adult rat dorsal root ganglion (DRG) neurons. The data suggest a coordinated regulatory axis linking Ang-II receptor signaling, TWIK1 expression, and nociceptive processing under both physiological conditions and in a model of neuropathic pain. These findings support a framework in which components of the renin–angiotensin system (RAS) may influence sensory neuron excitability, at least in part, through modulation of leak K^+^ conductances. This has potential implications for the use of angiotensin receptor blockers as therapeutic strategies in chronic pain.

Our results demonstrate that TWIK1 is broadly expressed across DRG neuronal subpopulations, with higher expression in large, heavily myelinated neurons, intermediate levels in medium-sized neurons, and lower expression in small, unmyelinated neurons. This distribution is consistent with previous reports (Mao et al., 2019; Mao et al., 2017; Pollema-Mays et al., 2013). In addition, TWIK1 showed stronger association with peptidergic neurons, as indicated by its positive correlation with trkA, and a weaker, negative correlation with IB4-binding neurons, in agreement with Mao et al. (2017). Differences reported across studies may reflect species-specific factors, antibody specificity, or genetic background. Importantly, the widespread expression of TWIK1 across neuronal classes suggests that its contribution to sensory processing is not restricted to a single functional subtype.

A key observation in this study is the co-localization of TWIK1 with both AT1R and AT2R, together with significant positive correlations between their expression levels. While co-localization does not establish direct interaction, it supports the possibility that TWIK1-expressing neurons are responsive to Ang-II signaling. This spatial association provides a structural basis for potential receptor–channel coupling mechanisms that could regulate neuronal excitability.

To explore this possibility, we examined whether pharmacological modulation of AT1R and AT2R alters TWIK1 expression. At the transcriptional level, combined blockade of both receptors increased TWIK1 mRNA following CCI, whereas single receptor inhibition did not produce a comparable effect. At the protein level, CCI induced a reduction in TWIK1 expression at later time points (7 and 14 days), consistent with previous studies (Mao et al., 2017; Pollema-Mays et al., 2013). Notably, this decrease was observed across neuronal subpopulations when comparing contralateral and ipsilateral sides, although only large neurons showed significant reductions relative to normal values. Given that different DRG neuron sizes encode distinct sensory modalities (Lawson, 2002; Lawson et al., 2019) these findings suggest that TWIK1 downregulation may affect multiple nociceptive pathways.

Pharmacological interventions revealed differential regulatory effects. The combination of telmisartan and PD123319 produced a robust increase in TWIK1 expression, particularly in small and medium neurons, exceeding levels observed in normal conditions. This pattern is consistent with a compensatory response aimed at restoring membrane stability following injury. Similar overcompensation phenomena have been described for potassium channels in conditions of altered excitability, as observed in chronic pain and epilepsy (Snowball and Schorge, 2015). In contrast, telmisartan alone increased TWIK1 expression in medium and large neurons, whereas PD123319 had minimal effects. These findings suggest that AT1R may play a dominant role in regulating TWIK1 expression, either through tonic inhibitory signaling or through indirect mechanisms involving downstream pathways.

Two non-mutually exclusive mechanisms may explain these observations. First, AT1R activation may suppress TWIK1 expression under pathological conditions, such that its blockade relieves this inhibition. Alternatively, combined inhibition of AT1R and AT2R may disrupt a broader signaling network, leading to enhanced TWIK1 expression. The limited effect of AT2R blockade alone argues against a simple model in which AT2R directly drives TWIK1 upregulation, suggesting instead a more complex interplay between receptor pathways.

Functionally, increased expression of K2P channels is generally associated with enhanced leak K^+^ currents and membrane hyperpolarization, thereby reducing neuronal excitability. In this context, restoration or upregulation of TWIK1 would be expected to counteract the hyperexcitability associated with neuropathic pain. Consistent with this idea, previous work has shown that TWIK1 overexpression can attenuate mechanical hypersensitivity (Mao et al., 2017). In the present study, pharmacological treatments that increased TWIK1 expression were associated with improvements in cold allodynia and mechanical hypersensitivity, although no effect was observed on spontaneous pain. This suggests that TWIK1 may preferentially influence stimulus-evoked rather than spontaneous nociceptive activity, in contrast to other K2P channels such as TREK2 (Acosta et al., 2014) and THIK1 (Haskins et al., 2017).

Behaviorally, both telmisartan and PD123319 produced partial and time-dependent anti-nociceptive effects, whereas the combination treatment resulted in more consistent relief of cold and mechanical hypersensitivity. Telmisartan showed effective relief of cold allodynia as soon as 3 days after CCI, an effect that continued throughout the assay. This agrees with previously published results in the same model and with a similar dose (Jaggi and Singh, 2011). PD123319 attenuated cold hypersensitivity at 3 and 7 days after CCI, but lost efficacy after that. This mimics other report in a similar model (Spinal Nerve Ligation, SNI) albeit the drug was administered by a different via (acute i.p. vs. continuous subcutaneous) (Shepherd et al., 2018). These findings align well with numerous reports in the literature showing that blocking either AT1 or AT2 receptors in CCI, SNL or SNI models of neuropathic pain, are effective at relieving mechanically-induced pain (Anand et al., 2015; Balogh et al., 2021; Chakrabarty et al., 2013; Muralidharan et al., 2014; Ranjbar et al., 2019; Smith and Muralidharan, 2015). The enhanced efficacy of the combination treatment suggests additive or synergistic effects, potentially reflecting simultaneous modulation of multiple signaling pathways.

An important aspect of this study is the analysis of AT1R and AT2R expression following CCI and pharmacological intervention. Contrary to the common assumption that receptor levels remain stable, we observed dynamic regulation of both receptors. AT1R expression decreased early after injury and subsequently increased above baseline levels, consistent with previous reports (Oroszova et al., 2017; Pavel et al., 2013). Telmisartan reduced AT1R expression after prolonged treatment, which may relate to its high binding affinity and prolonged receptor occupancy (Kakuta et al., 2005). In contrast, AT2R expression increased at later stages of CCI and was reduced by telmisartan and combination treatment. These findings indicate that both receptors are subject to regulation in response to injury and pharmacological modulation. Albeit these are interesting results, the controversy surrounding the localization and expression of AT2R in the peripheral nervous system requires a cautious interpretation.

While some studies, particularly in mice, report little to no neuronal Agtr2 expression and instead localize AT2R to infiltrating immune cells (e.g., macrophages), a substantial body of evidence supports its presence in DRG neurons in both rats and humans. Immunohistochemical and functional studies have identified AT2R in small-to medium-diameter nociceptive neurons, often co-localizing with TRPV1 and contributing to MAPK signaling pathways involved in pain processing (Anand et al., 2013; Benitez et al., 2017; Benitez et al., 2020; Regulski et al., 2015; Smith and Muralidharan, 2015). In addition, components of a local angiotensinergic system, including AT2R mRNA, have been detected in DRG tissue, with receptor expression reported to increase following nerve injury (Gallinat et al., 1998; Patil et al., 2010).

At the same time, non-neuronal expression of AT2R in satellite glial cells and infiltrating immune cells has also been described, suggesting that Ang II signaling may involve paracrine interactions within the DRG (Khan et al., 2017; Shepherd et al., 2018). Species differences may further contribute to these discrepancies, with neuronal AT2R expression more consistently observed in rat and human studies than in mice. In this context, the relatively low AT2R signal detected by Western blot in our study may reflect the low abundance of the receptor and methodological differences in sensitivity, rather than absence of expression. Nonetheless, we cannot exclude contributions from non-neuronal cells or potential limitations in antibody specificity.

The observed changes in receptor expression, together with behavioral outcomes, are consistent with a role of RAS signaling in neuroinflammation. AT1R activation is widely associated with pro-inflammatory processes (Balogh et al., 2021; Costa et al., 2014; Saavedra et al., 2011), whereas AT2R blockade have been linked to reduced immune cell infiltration and inflammatory signaling (Chakrabarty et al., 2013; Khan et al., 2017; Matavelli and Siragy, 2015; Shepherd and Mohapatra, 2018; Smith and Muralidharan, 2015). Furthermore, there is evidence of upregulation of AT2R in nociceptors during CFA-induced inflammation (Benitez et al., 2020). In agreement with this, we observed that pharmacological treatments reduced circulating levels of pro-inflammatory cytokines, including TNF-α, IL-1β, and IL-6, consistent with previous reports (Jancalek et al., 2010; Jancalek et al., 2011; Okamoto et al., 2001). These systemic anti-inflammatory effects may contribute to the modulation of TWIK1 expression and nociceptive signaling.

At the level of the DRG, satellite glial cells (SGCs) represent a key component of the inflammatory microenvironment. Following nerve injury, SGC activation is characterized by increased GFAP expression, cellular hypertrophy, and enhanced gap junction coupling, all of which have been linked to pain sensitization (Du et al., 2023; Ji et al., 2016; McGinnis and Ji, 2023; Qiu et al., 2024). In the present study, only the combination treatment reduced SGC activation, suggesting that simultaneous inhibition of AT1R and AT2R is required to effectively modulate local glial responses. This may help explain the superior behavioral efficacy of the combined treatment.

Taken together, the data support a model in which CCI-induced increases in Ang-II signaling promote neuroinflammation and reduce TWIK1 expression, thereby enhancing neuronal excitability. Pharmacological blockade of AT1R and AT2R attenuates this inflammatory response and restores TWIK1 expression. Supporting this model, *in vitro* exposure of DRG neurons to inflammatory mediators reduced TWIK1 mRNA levels, providing direct evidence that inflammatory signals can regulate TWIK1 expression at the transcriptional level.

In summary, this study identifies a link between RAS signaling, inflammatory processes, and regulation of a K2P channel in sensory neurons. The combined modulation of receptor activity, ion channel expression, and inflammatory state may underlie the observed anti-nociceptive effects. These findings provide a basis for further investigation into the therapeutic potential of targeting Ang-II receptors, alone or in combination, in chronic pain conditions. This aligns with the modern trend of repurposing well-known and fully characterized drugs like ARBs to treat other illnesses, such a refractory pathological pain (Chatzipieris et al., 2025).

## 5 Limitations and further directions of the study

Although this study was conducted in accordance with current recommendations for the inclusion of sex as a biological variable and focused on female rats due to the higher prevalence of chronic pain in women, we acknowledge that the exclusive use of one sex represents a limitation. Given well-documented sex differences in pain processing and in responses to pharmacological modulation of nociceptive pathways (Mills et al., 2019; Presto et al., 2022; Stephens et al., 2019), it is possible that AT1R/AT2R signaling and its regulation of TWIK1 may not be fully identical in males. Therefore, future studies including both sexes will be important to determine the extent to which these findings can be generalized across sexes and to identify potential sex-specific mechanisms.

Despite the controversy surrounding the precise cellular localization of AT2R, our results support a model in which TWIK1 downregulation is mediated by pro-inflammatory signaling mechanisms. In this regard, cytokines released from non-neuronal cells, such as infiltrating macrophages or satellite glial cells, may contribute to the observed effects, consistent with a paracrine mode of action within the DRG. Therefore, our main findings remain compatible with both neuronal and non-neuronal AT2R localization, while further studies will be required to resolve this issue definitively.

To further strengthen the evidence for a causal role of TWIK1 in the pathway initiated by Ang-II upregulation following nerve injury or inflammation, future studies employing targeted gene manipulation approaches (e.g., AAV-mediated knockdown or overexpression in DRG neurons) would be of considerable value. In addition, the development of selective pharmacological tools capable of positively modulating TWIK1 expression or activity (currently lacking) would provide an important complementary strategy to validate its functional role in neuropathic pain and may also offer potential therapeutic avenues.

## Data Availability

The raw data supporting the conclusions of this article will be made available by the authors, without undue reservation.
