# Supplementary material for: Angiotensin II type 1 and type 2 receptors modulate TWIK1 channel expression and pain sensitivity in a rat model of neuropathic pain
Source: Front Pharmacol. 2026 May 21;17:1833813. doi: 10.3389/fphar.2026.1833813 (PMC13233714; doi:10.3389/fphar.2026.1833813)
Supplement: Supplementary file 1 [file Supplementaryfile1.docx]

Supplementary Material


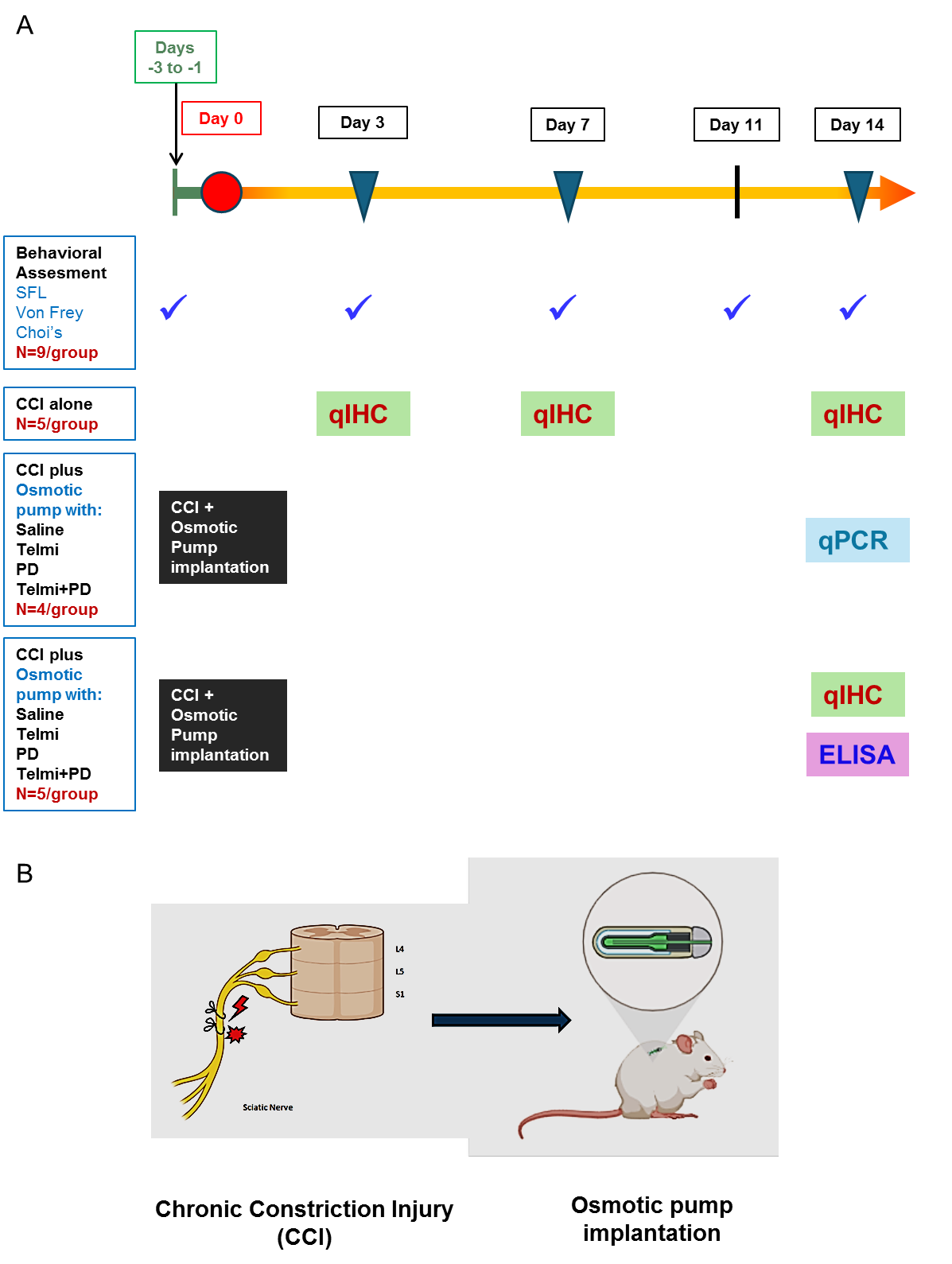


**Supplementary Figure 1. (A)** Diagram of experimental desugn including the simple size for each treatment group and techniques carried out. **(B)** Illustration showing the modified version of the chronic constriction injury used in this study, followed by implantation of ALZET osmotic pumps.


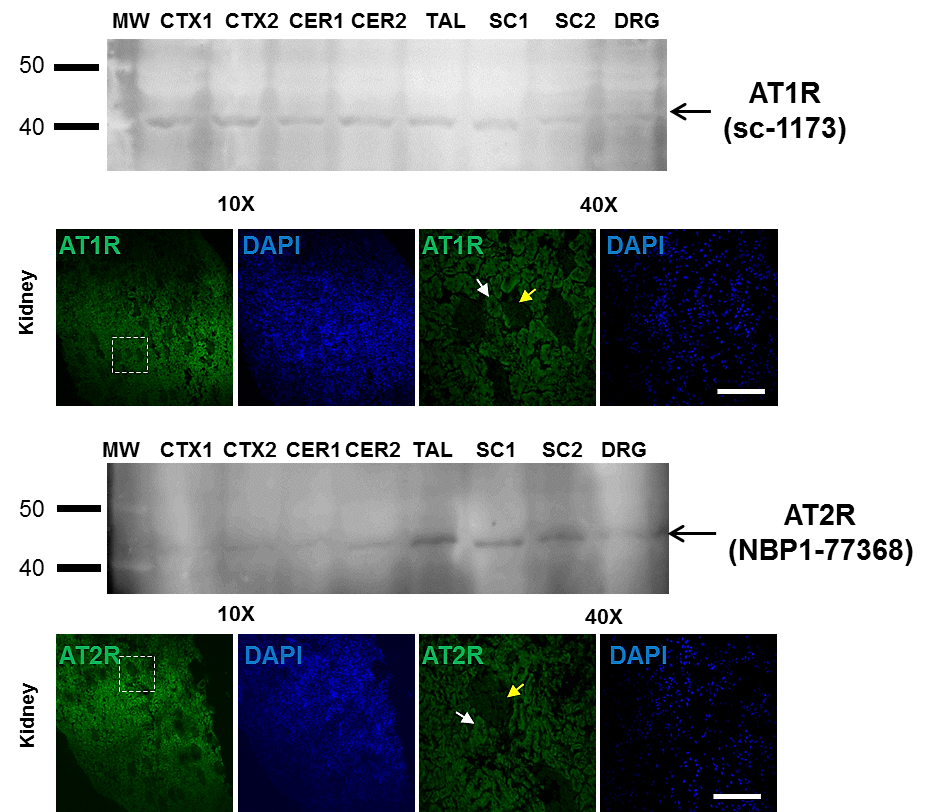


**Supplementary Figure 2.** Antibodies against AT1R and AT2R were characterized by Western blot (WB) and immunohistochemistry. Homogenates from the central nervous system (cortex, cerebellum, thalamus, and spinal cord) and the peripheral nervous system (PNS) of adult rats were analyzed by WB. To further establish antibody specificity by immunohistochemistry, rat kidney tissue was used because its expression pattern is well characterized in the literature. Both antibodies used in this study showed an expression pattern similar to that previously described in renal tissue, mainly labeling the renal tubules (white arrow) and, to a lesser extent, the renal glomeruli (yellow arrow). Scale bar 50 µm.


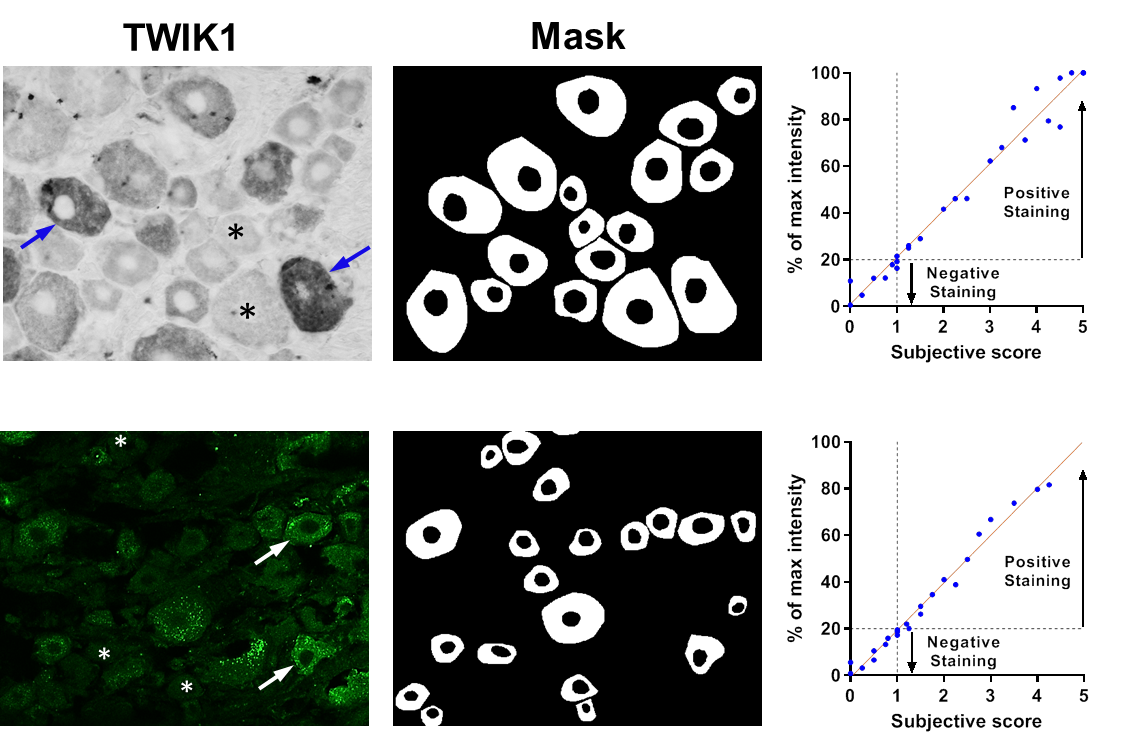


**Supplementary Figure 3**. Examples of quantitative vs. qualitative scoring used for analysis of ABC/DAB staining (top) and immunofluorescence staining (bottom).


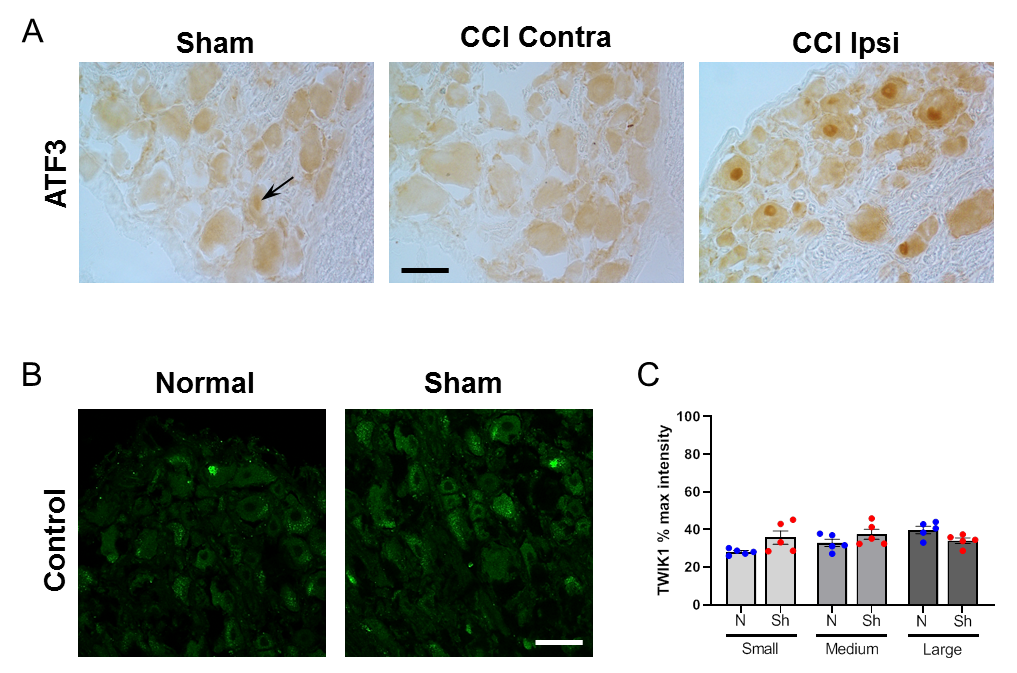


**Supplementary Figure 4**. **(A)** Characterization of the damage induced to DRG neuronal somata triggered by CCI as ascertained with nuclear expression of the transcription factor ATF-3. **(B)** Representative images of normal and sham L5 DRG sections stained against TWIK1. Scale bar 50 µm. **(C)** Scatter bar plot showing that the % of maximum intensity of the K2P channel does not change significantly between sham and normal, in all the different subpopulations of neurons as classed by size.
